# The complete chloroplast genome of a shrub species, *Smilax glabra* (Smilacaceae) from Guangdong, China

**DOI:** 10.1080/23802359.2021.1979431

**Published:** 2021-09-27

**Authors:** Yi Zhang, Ying Liu, Qijin Ge, Kai Han, Chengcheng Shi, Xin Liu

**Affiliations:** a School of nursing, Chongqing Medical and Pharmaceutical College, Chongqing, P. R. China; b BGI Education Center, University of Chinese Academy of Sciences, Shenzhen, P. R. China; c BGI-Qingdao, BGI-Shenzhen, Qingdao, P. R. China; d School of Future Technology, University of Chinese Academy of Sciences, Beijing, P. R. China; e BGI-Fuyang, BGI-Shenzhen, Fuyang, P. R. China

**Keywords:** Complete chloroplast genome, Liliales, phylogeny, Smilacaceae, *Smilax glabra*

## Abstract

*Smilax glabra* is a perennial woody scandent shrub, of which the dried aerial tuber has been used as Chinese medicine. Here, we sequenced *S. glabra* and assembled its complete chloroplast (cp) genome. The genome is 157,889 bp in length and has a typical quadripartite structure. We annotated 131 genes, of which 84 were protein-coding genes, 37 were tRNAs and 8 were rRNA genes. Phylogenetic analysis of this genome with 26 representatives Liliales fully resolved *S. glabr*a in a clade with *S. china*. The phylogenetic tree we constructed is largely consistent with recently published phylogenetic trees using both complete chloroplast genomes and marker gene sequences.

*Smilax glabra* Roxb. is classified in the Smilacaceae and is commonly known as Chinaroot or sarsaparilla. It is a shrub and inhabits forests, thickets, thinly forested slopes along valleys and riverbanks (Wu and Raven 2000). It is distributed widely in southern China, Southeast Asia and South Asia (Wu and Raven 2000). The aerial tubers of *S. glabra* might contain metabolites with antibacterial and anti-inflammatory features and thus have been used as Chinese medicine for abscesses, arthritis rheumatism, syphilis and other diseases (GBIF 2020). With abundant active compounds including dihydro-flavonol glycosides and flavanonol rhamnoside (Chen et al. 2002; Yuan et al. 2004), tuberlike rhizomes of *S. glabra* are also used to treat cancer (Sa et al. 2008). Furthermore, it can also be used as an antidote for mercury poisoning (Commission, Chinese Pharmacopoeia 2015). Previous research on *S. glabra* focused on depicting its secondary metabolites, and possible functions of these secondary metabolites, as cited above. Markers on the chloroplast genome were sequenced to analyze the relationship among different individuals of *S. glabra* and compare them to other related species (Li et al. 2011; Wang et al. 2014). Despite these previous studies on *S. glabra*, its evolutionary history, as well as its genetic content, remained largely unexplored. Here, we report the complete chloroplast of *S. glabra* to contribute to the systematics and bioinformatics of this species.

The sample was collected in Guangdong, China (N22°35.504′, E114°16.283′) and a specimen (plant tissues and seeds) were deposited at the Herbarium of China National GeneBank (https://db.cngb.org/brc/plant/NGBYW00012, collected by Xuebing Wang et al., email: P_brc@cngb.org) under the voucher number NGB0003149. DNA was extracted from young leaves and the sequencing was carried out using a BGISEQ-500 sequencer (BGI Qingdao, Qingdao, China). In total, ∼10 Gbp reads were generated and ∼0.5 Gb of data were randomly extracted for the chloroplast genome assembly. NOVOplasty (Dierckxsens et al. 2017) and MITObim (Hahn et al. 2013) were used to assemble the chloroplast genome (setting the parameters of K-mer 29 in NOVOplasty and using *Smilax china* chloroplast genome as the reference genome in both software). The complete chloroplast genome was integrated according to the alignment of the two assemblies (by NOVOplasty and MITObim, accordingly) using MAFFT (Katoh et al. 2009). The chloroplast genome was annotated using GeSeq (Tillich et al. 2017).

The chloroplast genome of *S. glabra* is a single circular DNA sequence with a length of 157,889 bp. It has a typical quadripartite structure, with a large single copy (LSC) region of 85,280 bp, a small single copy (SSC) region of 18,685 bp, and the two inverted repeat (IR) regions of 26,962 bp. The IR regions had higher GC content (42.6%) than the LSC and SSC regions (34.9% and 31.0%, respectively). The genome contains 131 genes, including 84 protein-coding, 8 ribosomal RNA (rRNA), and 37 transfer RNA (tRNA) genes.

The phylogenetic tree was constructed using 25 representative chloroplast genomes from 25 genera of Liliales, along with three closely related species designed as outgroup taxa (*Carludovica palmata* from Pandanales, *Calanthe sylvatica* from Asparagales, and *Polygonatum cyrtonema* from Asparagales). The coding sequences were used to obtain 65 gene clusters. The gene clusters were aligned using MAFFT (Katoh et al. 2009) and the tree inferred with RAxML (Stamatakis 2014) (using a model of GTR-GAMMA-I, and 1,000 bootstrap replicates). The phylogenetic tree constructed (Figure 1) reflected the relationship of families within the order of Liliales. The family of Liliaceae, which has the most species in this order, was closely related with Smilacaceae, to which *S. glabra* is classified. This is consistent with the phylogenetic tree constructed using both complete chloroplast genomes and marker gene sequences recently published (Do et al. 2020). The Melanthiaceae was a sister group to Liliaceae and Smilacaceae families. The Alstroemeriaceae and Colchicaceae were positioned in a clade sister to the Liliaceae, Smilacaceae and Melanthiaceae. Finally, we found the Campynemataceae (with the representative species of *Campynema lineare*) to occupy a basal lineage in the Liliales clade.

**Figure 1.**
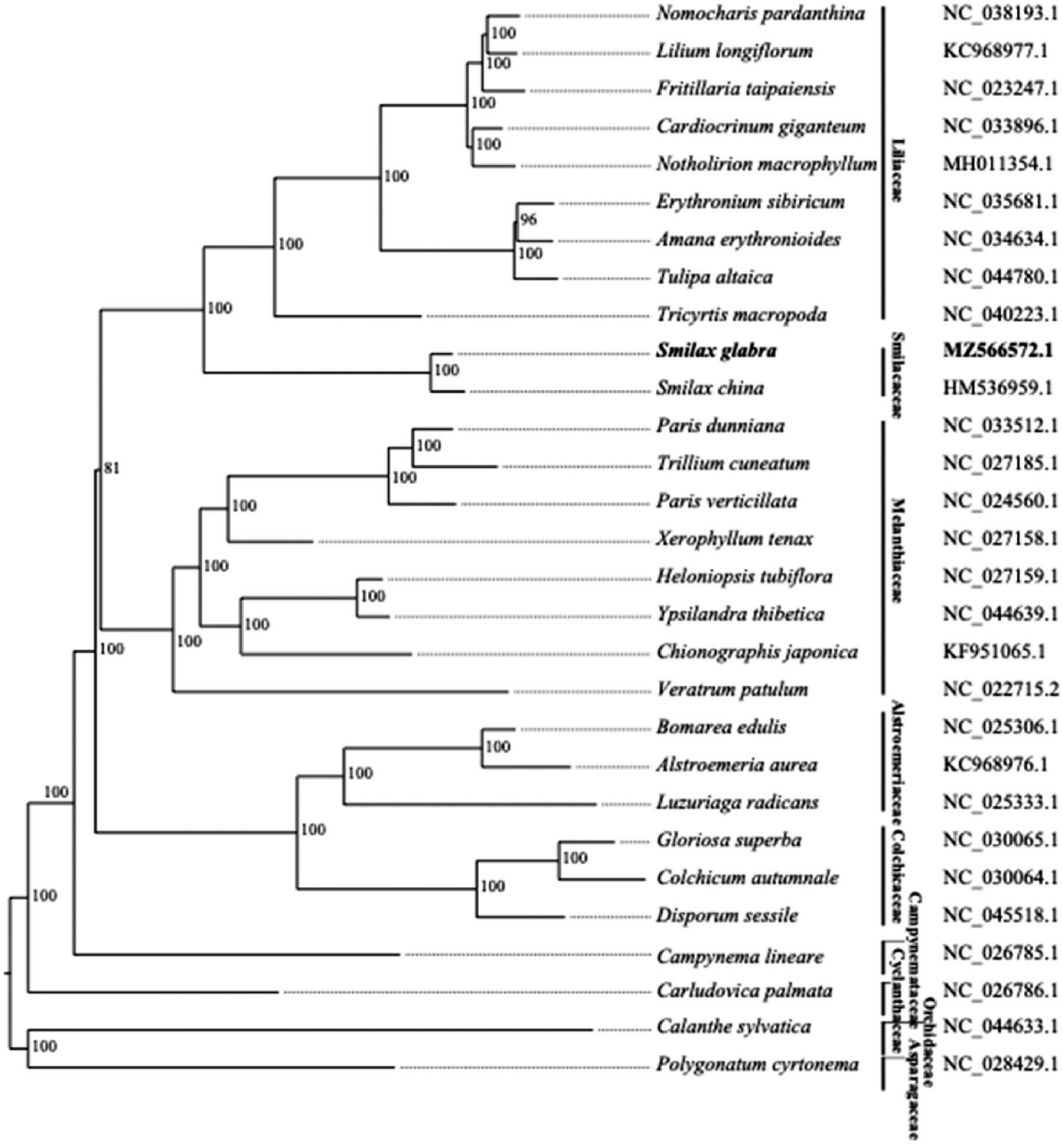
Phylogenetic tree of Liliales species based on genes on chloroplast. 25 representative species for 25 genera of Liliales, along with 3 outgroup species (the accession numbers were indicated on the right) and *Smilax glabra*, are shown in the phylogenetic tree with the numbers indicating the bootstrap value of each clade based on 1000 replicates.

## Data Availability

The genome sequence data that support the findings of this study are openly available in GenBank of NCBI and CNGBdb of China National Genebank (CNGB). In NCBI, the data is deposited in GenBank of NCBI at (https://www.ncbi.nlm.nih.gov/nuccore/MZ566572) under the accession no. MZ566572. The associated BioProject, SRA, and Bio-Sample numbers are PRJNA746120, SRS9463563, and SAMN20181735 respectively. In CNGBdb, the data can be found under the project CNP0001886 (https://db.cngb.org/search/project/CNP0001886/).
